# Magmatic domes and the initiation of oceanic processes at the Norwegian margin

**DOI:** 10.1038/s41598-025-18924-9

**Published:** 2025-10-08

**Authors:** Gwenn Peron-Pinvidic

**Affiliations:** Department of Geosciences, Norwegian University of Science and Technology (NTNU), Trondheim, Norway

**Keywords:** Rifting, Rifted margin, Breakup, Oceanic ridge, Geodynamics, Tectonics

## Abstract

The Wilson-cycle is a conceptual model accounting for the assembly, dismembering, and separation of continental masses on Earth. Yet, beyond the widely used concept, the mechanics of the final breakup of continents as a fundamental step remain weakly constrained. The beginning of oceanic accretion is nowhere firmly observed. It is usually reported as corresponding to gradual structural and/or magmatic variations, but no specific geometry has been identified as witnessing the actual onset of an oceanic spreading ridge. Based on a deep penetrating high resolution seismic reflection dataset, this contribution reports new geometries from the outer domain of the Mid-Norwegian rifted margin. A series of systematic magma-related features is observed and described within the context of the lithospheric breakup. The reported geometries may help better understand the transition from rift to drift processes, including possible domes of accumulated frozen magma, fractures and dikes radiating systems, intrusives and lava flows.

## Introduction

‘Breakup’ is a widely used term that designates the end of rifting and the beginning of oceanic drifting. Implicitly, it refers to a unique, instantaneous, and localized process that ‘breaks’ pre-existing settings - the continental lithosphere - to produce a new oceanic lithosphere. The simplicity and straightforward definition of the word contributed to its success. When the term was introduced in the 1950-60’s^1^ to distinguish domains floored by oceanic and continental crust, no observation was available from the related offshore frontier areas. So, the limit was based on remote sensing dataset where magnetic anomalies were identified as the first organized stripes of oceanic crust^2^. With today’s available datasets and improved knowledge of geodynamic processes, the existence of a more accurate definition should be expected. However, this is not the case, most probably because no consensus has been reached on the architecture of the domains that record this geological process, i.e. the distal rifted margins and first domains of oceanic crust.

**Fig. 1:**
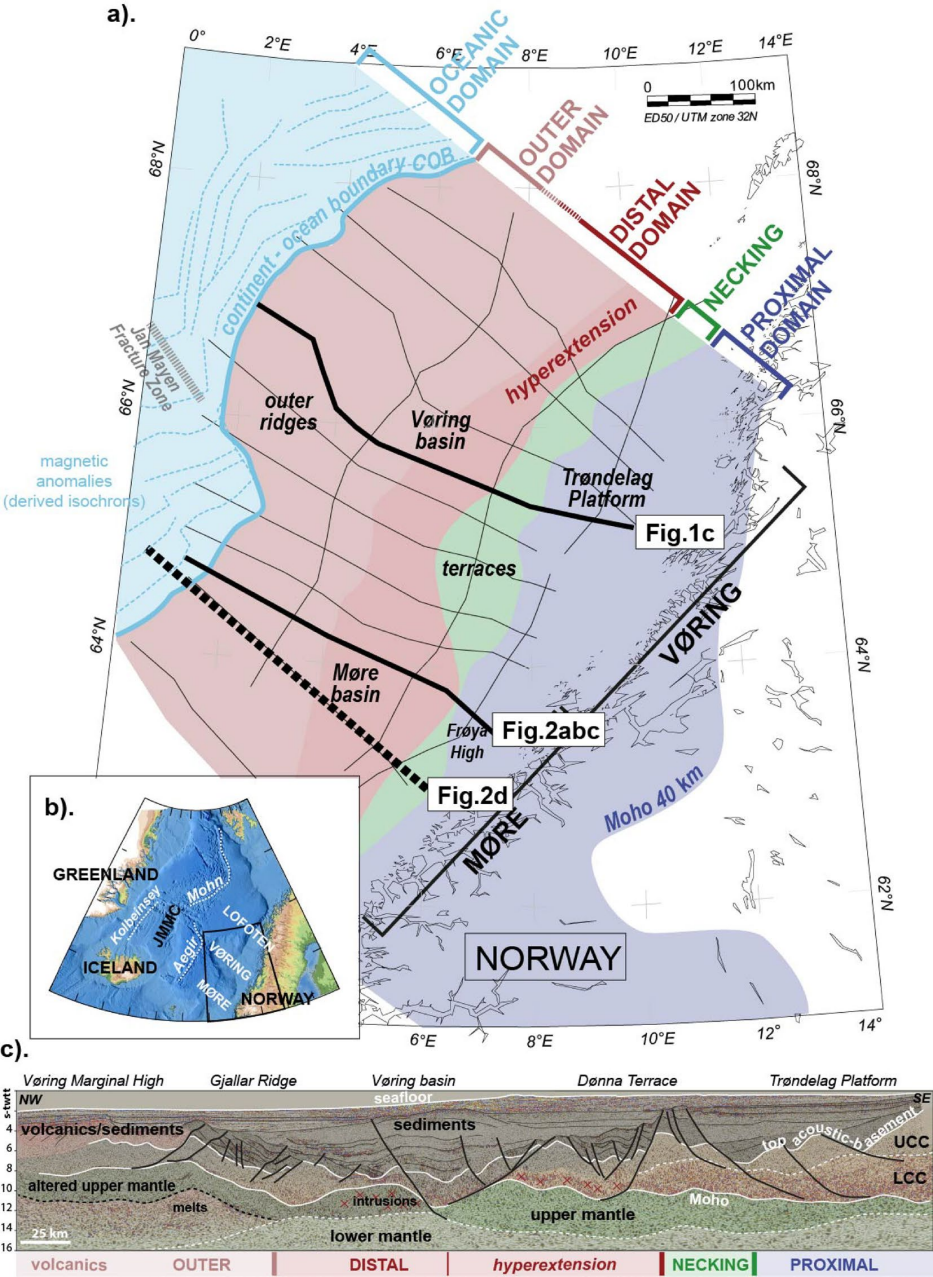
**a**). Map of the study area. The thin black lines show the profiles of the seismic reflection Geoex MCG RDI19 dataset used in the study. The coloured polygons represent the different margin structural domains as defined by Peron-Pinvidic et al. (2022). []The thick black lines highlight the profiles presented at the bottom of the figure (Fig. 1**c**.) and in Fig. 2. The dashed blue lines West of the COB represent the derived oceanic isochrons based on magnetic anomalies picks by Gaina et al.^35^ **b**). Bathymetric and topographic map of the Northeast Atlantic region localizing the study area. JMMC Jan Mayen Micro Continent **c**). Seismic reflection profile selected as representative of the architecture of the Mid-Norwegian margin. The red crosses show the probable presence of magmatic intrusions (based on potential-field modelling). From Peron-Pinvidic et al. (2022). Courtesy of Geoex MCG.

Structurally, rifted margins typically show a seaward arrangement of characteristic entities such as the presence of shallow-water platforms and deeper basin areas (the ‘proximal’ and ‘distal’ margins), domains where continental crust is more abruptly thinned (the ‘necking’), and domains where mantle can be exhumed (Fig. 1). A consensus has been reached that these geometries describe most rifted margins worldwide and are the result of a multiphase evolution^3^^4^^5^^6^;;;. Oceanward, the oceanic domain encompasses the areas of newly accreted igneous basement that are strictly oceanic in origin and represent steady-state formation of oceanic crust^7^^8^;. The COB (continent-ocean boundary) then refers to the demarcation, in time and space, between these two fundamental domains and used to be summarized as an abrupt boundary^9^.

Although these first-order definitions sound straightforward, the distinction between the oceanic domain and the distal margin may not be that simple. From studies of slow-spreading ridges, it is currently recognised that oceanic crust forms not only through magmatic addition but also through tectonic extensional processes^10^^11^^12^^13^;;;. The structural similarities between oceanic and continental core complexes are frequently highlighted^14^^15^;. These observations open the question of what the intrinsic origin and nature of the first domains of oceanic crust are, as opposed to distal rifted margins. In these magma-poor environments, the COB concept is questioned and recognized as being oversimplified^9^^16^;. The existence of ‘transition zones’ where structural and/or magmatic processes gradually change from rifting to drifting is regularly advocated - these are often called ‘proto-oceanic’ or ‘hybrid crust’^17^^18^;. To better represent the various lithologies and related geological processes, additional definitions have been introduced, such as the landward limit of the oceanic crust (LaLOC^19^^20^;; and the edge of continental crust (ECC^21^;. Recently, based on the southern part of the North Atlantic Ocean and wide-angle refraction data, Welford^22^ further analysed these specific regions. Rather than trying to define limits, she defined geographical areas regardless of their basement components. She characterized COTZs (Continent-Ocean Transition Zones) and showed that they encompass various lithologies including thin continental, exhumed mantle and thin oceanic crust.

In the case of magma-rich margins, the limit between continental-derived and oceanic-derived basement is a priori more obvious because of the related magmatic material. Magmatic features usually have noticeable geophysical signals - whatever the method, with, e.g., lava flows, sills, and seaward-dipping reflectors (SDR) obvious on seismic reflection profiles; magmatic underplating and/or intrusions leading to overthicken crust visible on wide-angle refraction and gravity data; and various types of extrusive or intrusive features that can hold strong magnetic signals. In addition, in certain cases, the related volumes are very important and thus outstanding on all datasets (e.g., the Pelotas margin^23^;. In these magmatic environments, the COB is standardly interpreted as related to the emplacement of the SDR packages, with a limit defined either at the top or at the bottom of the wedge of reflectors. Based on the identification of various types of SDRs, either tectonic- or magmatic- driven, McDermott^24^ further proposed a geographical delimitation mapped at the limit between these distinct categories.

Lately, hybrid type of rifted margins have been reported^6^; e.g., the Santos basin^25^,, what opens the door to even more basement-types limits definitions. The Mid-Norwegian rift system can arguably be listed under that category^26^. However, there, historically, the distinction between continental and oceanic domains has been solely based on a COB-type of approach, which is method-dependent. The result is that, depending on the dataset and definition that is picked (gravity or magnetic grids, seismic reflection, seismic refraction), interpreters defined very different COBs, potentially up to dozens of km apart [e.g^27^^28^^29^^30^^31^^32^^33^^34^.,;;;;;;;.

Based on the above, it can be concluded that, although major improvement in our knowledge allowed the development of more accurate interpretations and models, COTZs remain domains where our understanding still fails to provide consensual definitions. COB and COTZ related limits are method- and rifted margin type- dependent.

This contribution reports new observations from the Møre and Vøring rifted margins located offshore Norway, in the North-East Atlantic (Fig. 1), and proposes to enrich our catalogue of observations, describing the presence of specific geometries at lower basement depths in the outer margin. The reported structures are discussed and interpreted to be related to the evolving magmatic activity of the outer margin during the rift to drift transitional stage.

## Study site

This contribution reports observations from the Møre and Vøring segments of the Mid-Norwegian rifted margin located offshore Norway, in the North-East Atlantic (Fig. 1). Structurally, this rifted system is categorized as a wide, sediment-rich and magma-rich margin^36^. The global architecture corresponds to the juxtaposition of distinct structural domains (proximal, necking, distal, outer; Fig. 1), shaped by distinct deformation phases (reactivation, stretching, thinning, hyperextension, exhumation, oceanization)^26^. Chronologically, the early rift phases (pre-Mesozoic, Permian, Early Triassic) shaped the rift basins that today are recognized in the proximal settings (dark blue colour on Fig. 1a, c and e.g. the Trøndelag Platform). In Jurassic times, deformation migrated oceanwards and focused on specific regions, creating the necking domain, with for instance the Halten and Dønna terraces (green colour on Fig. 1a and c). Then extension further migrated oceanwards by Cretaceous times, shaping the distal margin (reddish colours on Fig. 1a and c; the Vøring and Møre Basins). And during Late Cretaceous - Paleocene, a last extensional episode affected the outer ridges and sub-basin system at the edge of the COB line (pinkish colour on Fig. 1a and c) leading to the construction of the magma-dominated outer domain. Continental breakup between the Eurasian and Greenland plates occurred in Early Eocene time^37^. A last phase of rifting affected the area in Cenozoic times, on the East Greenland side, leading to the isolation of the Jan Mayen microcontinent^34,38,39^ and to the formation of the today’s active Kolbeinsey oceanic ridge, letting the Aegir ridge inactive (Fig. 1b).

Thus, the distal and outer domains - focus of this contribution - record a complex tectono-magmatic evolution from early Jurassic extension to Late Cretaceous - Paleocene hyperextension and continental breakup. Structurally, these domains show progressive thinning of the continental crust, development of detachment faults, exhumation of subcontinental mantle, and extensive magmatic activity. The resulting geometries are complex, and their interpretation is often disputed.

## Results

### Observations

**Fig. 2:**
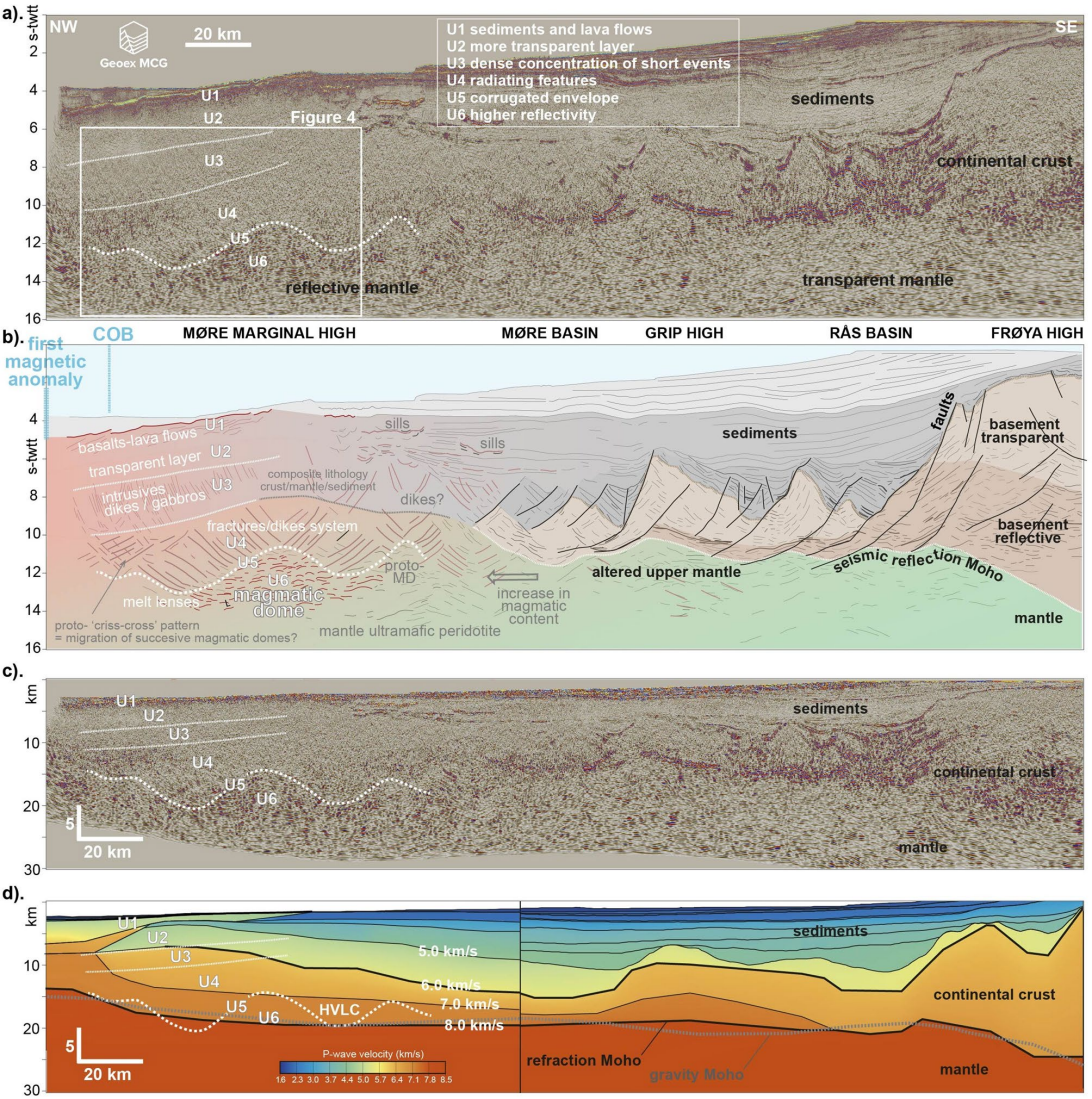
Geoex MCG deep penetrating long-offset seismic reflection profile imaging the architecture of the Møre rifted margin. **a**). Without interpretation. **b**). Interpreted time version. **c**). Depth converted version. MD: magma-dome. COB: continent-ocean boundary. U1-6: the various units defined based on their distinct seismic reflection facies and geometries. d). Composite seismic refraction profile compiled by Funck et al. (2017). The dashed grey line shows the gravity Moho modelled by Haase et al. (2017), and the white lines and labels refer to the observations discussed in this contribution. HVLC: high-velocity lower crust. See Fig. 1a for location.

**Fig. 3:**
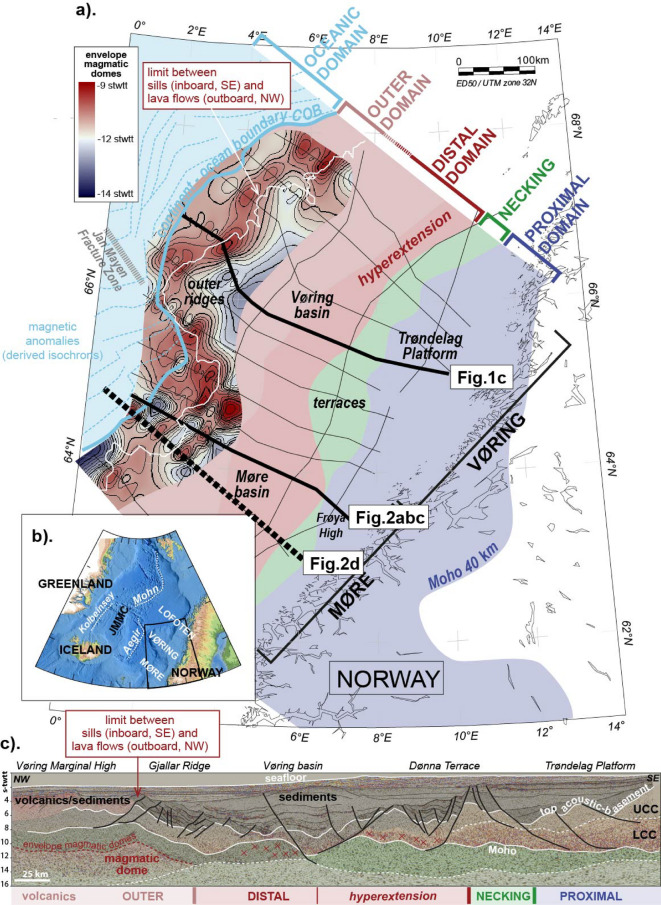
Similar Figure as Fig. 1 with additional information related to the identified domes. The aim is to underline the novelty of the observations reported here. a). Similar to Fig. 1. Novelty: The gridded surface (red-white-blue colours) displayed on the outer domain of the margin corresponds to the top envelope capping the potential proto-domes (U5 surface on Fig. 2). The thin white line running within the outer domain represents the limit between the sills (continentward) and the lava flows (oceanward)^41^. b). Same as Fig. 1: regional inset to localize the study area. JMMC Jan Mayen Micro Continent c). Similar to Fig. 1. Novelty: the interpretation in the outer margin is highlighted in red to ease the identification of the domal geometries. Modified from Peron-Pinvidic et al. (2022). Courtesy of Geoex MCG.

Figure 2 presents a typical seismic reflection profile issued from the Geoex MCG RDI19 long offset deep towed 16 s-twtt (second two-way-travel-time) dataset. The full margin architecture is imaged including top basement, Moho and upper mantle, from the most proximal regions to the East to the transition to the oceanic crust to the West (Figs. 1 and 2a.). The profile on Fig. 2a shows the general architecture of the Møre margin, from the thick continental crust of the Frøya High in the South-East to a series of faulted and tilted basement blocks under the Rås and Møre basins, and volcanics in the Møre Marginal High area to the North-West, next to the COB (Fig. 2ab). Figure 2d shows a composite seismic refraction model^40^ located nearby the Fig. 2a profile, aiming at comparing the observations reported here with existing other dataset Fig. 3

**Fig. 4:**
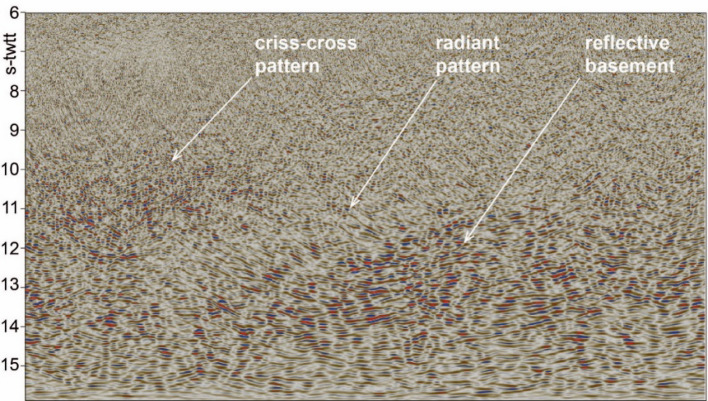
Zoom without interpretation of the outer margin domain discussed in this contribution. See Figure 2a. for location.

Focus is set here on the margin outer domain. In this area, six elements are systematically observed on all profiles. These are labelled U1 to U6 (U for Units; Figs. 2a and 4). The first observation relates to the seismic reflectivity at lower crustal levels (> 10 s-twtt), which significantly increases oceanwards, from a relatively less reflective mantle in the south-east, underneath the continental crust remnants, to a more reflective mantle to the north-west, under the Møre Marginal High (Fig. 2a). The change in seismic reflectivity is accompanied by the presence of multiple short reflectors of medium to high amplitude (U6). These form a consistent unit at depth and are capped by a thin layer of more transparent seismic facies at 10–12 s-twtt depth. U5 is not a unit per se but rather a capping envelope (U5 dashed white line on Fig. 2abcd) that presents, at the regional scale, a corrugated shape with regular dome-like geometries of various amplitudes (10–30 km). Similar observations are made on all profiles of the dataset. Figure 3a displays a gridded surface of the U5 envelope. The resulting surface is biased by the low number of profiles and the distance between the observations, resulting in some gridding artefacts. Although imperfect, the surface does give a sense of the regional extent of the related geological objects. Above the envelope, multiple sharp short reflectors of low to medium amplitude delineate an ‘escaping’-like geometry, with the various reflectors radiating outward from the underlying reflective dome (U4). Then, three distinct units overlie the radiating complex: a unit characterized by a dense concentration of short and thin reflectors of low amplitude (U3), a globally more transparent and structureless unit without specific or systematic geometry (U2), and the shallow reflective lava flows, probably intercalated with sedimentary layers (U1) (Figs. 2ab and 4). Figure 4 is an extract of the Fig. 2a seismic profile. It offers a zoom without interpretation of the outer margin domain discussed in this contribution. It clearly shows the distinct radiant and criss-cross patterns corresponding to the unit U4 (Fig. 3abc).

## Interpretation

The above-summarized geometries are typical from the outer domain of both the Møre and Vøring margin segments. Given the location within the distal margin, the related structures are probably associated with changes in the final rifting and, maybe, early drifting processes.

Additional observations confirm the connection between the U1-6 units described here and the magmatic history of the distal and outer margin:

1. The Fig. 3a map displays a thin white line in the margin outer domain corresponding to the limit between sills and lava flows^41^. The sills are observed and mapped on the continentward side of the line, while lava flows extend oceanward. The demarcation line nicely matches the U5-U6 main dome locations (see gridded envelope on Fig. 3a).
2. Figure 2d shows a composite seismic refraction model^40^ located nearby the Fig. 2a seismic reflection profile, which shows a good match between the high-velocity lower crust (HVLC on Fig. 2d) and the U5 and U6 units. HVLC bodies are usually interpreted in terms of magmatic additions in the form of large-scale underplating or massive infiltration and intrusion [e.g^42^.,. Although the seismic refraction data does not capture the geometrical details observable on the seismic reflection, there is a good match between the HVLC body and the units U5 and U6 outlined here.

So, in addition to the spatial correspondence between the domes and the magmatic events extruded and/or intruded in the distal and outer margin (point 1.), the observations from other geophysical data also point to a magmatic interpretation of the domes and related U-series features (point 2.). The question is then to understand if these domes could be related to the actual rift-to-drift transition and thus initiation of the oceanic accretion processes.

In comparison to the available data on rifts and rifted margins, few seismic reflection data are available on oceanic spreading systems. Consequently, the facies and characteristics of the seismic-reflection constituting elements of an oceanic ridge are relatively less well constrained. However, some local key observations have been reported from various settings, including ultra-slow, slow and fast accreting environments. Their analysis document a peculiar facies, often seismically transparent and structureless^43^^44^;. In some cases, a three-layers architecture is claimed^45^^46^; corresponding to the original 1972 Penrose Conference petrology-based definition. In that case, the upper basalt unit can span a range of seismic facies from transparent to highly reflective and structured, including lava flows, intrusives and SDR-type geometries^47^^48^^43^;;. The underlying sheeted dyke complex correspond to crisscrossing high angle reflectors^46^, and deeper massive gabbros and/or ultramafic units are usually reported as being majorly transparent and structureless^44^. In some cases, the presence of short bright reflectors is reported and interpreted as imaging the top of isolated axial magma chambers and possible off-axis frozen melt bodies emplaced at different basement levels; like at the East Pacific Rise^49^^50^;, Juan de Fuca Ridge^51^, Galapagos spreading center^52^, Mid-Atlantic Ridge^53^, South China Sea^54^.

Based on the geographical and structural connection between the U-series and the magmatic events affecting the outer margin at breakup time, the geometries described here (U5-6) may also be interpreted as magmatic domes (Fig. 2b). Within that context, the corrugated envelope (U5) capping the observed culminations of high reflectivity (U6) is interpreted as corresponding to the limit between the dominantly brittle (above) and dominantly ductile (below) mantle (at formation time). Nemčok and Frost^55^ have interpreted a similar boundary on their dataset of an extinct oceanic ridge axis in the Caribbean Sea. In their context, the corrugated surface facilitates the melts’ transport with fluids circulating towards the high points of the surface (the domes), and focusing there in the form of isolated pockets of melts. In the study area presented here, the domes correspond to high-velocity lower basement layers (Fig. 2d), which may be explained by the presence of similar accumulations of (now frozen) magma bodies. Crystallized magma sills/lenses injected in the mantle seem to be a reasonable hypothesis given the outer margin rift-to-drift context. Geographically, the assumed magmatic domes align in the outer margin domain sub-parallel to the COB line, in direct link to the magmatic extrusive events affecting the outer margin (above description and Fig. 1a). This alignment is interpreted as pointing to the existence of a SW-NE axis focusing magma at the rift scale, and not only local domes. However, a denser dataset would be necessary to argue between the multiple isolated domes vs. one-axis hypotheses. The proposed U5 surface on Fig. 3a does not allow this distinction given the gridding artifacts.

It is noted that the source of reflectivity within the interpreted magma domes needs further investigation. Studies of mid-ocean ridges indicate that former melt lenses exhibit no obvious reflectivity once the crust has cooled and drifted away from the ridge. This absence of or low reflectivity is attributed to the lack of impedance contrast between the cooled gabbroic melt lenses and the surrounding gabbro host rock (above references). However, some studies reported the existence of seismic reflectivity within the uppermost mantle/Moho transition zone and interpreted these as magmatic intrusions^56^ or lenses of gabbroic composition and melt accumulations within mantle^49^.

## Discussion

**Fig. 5:**
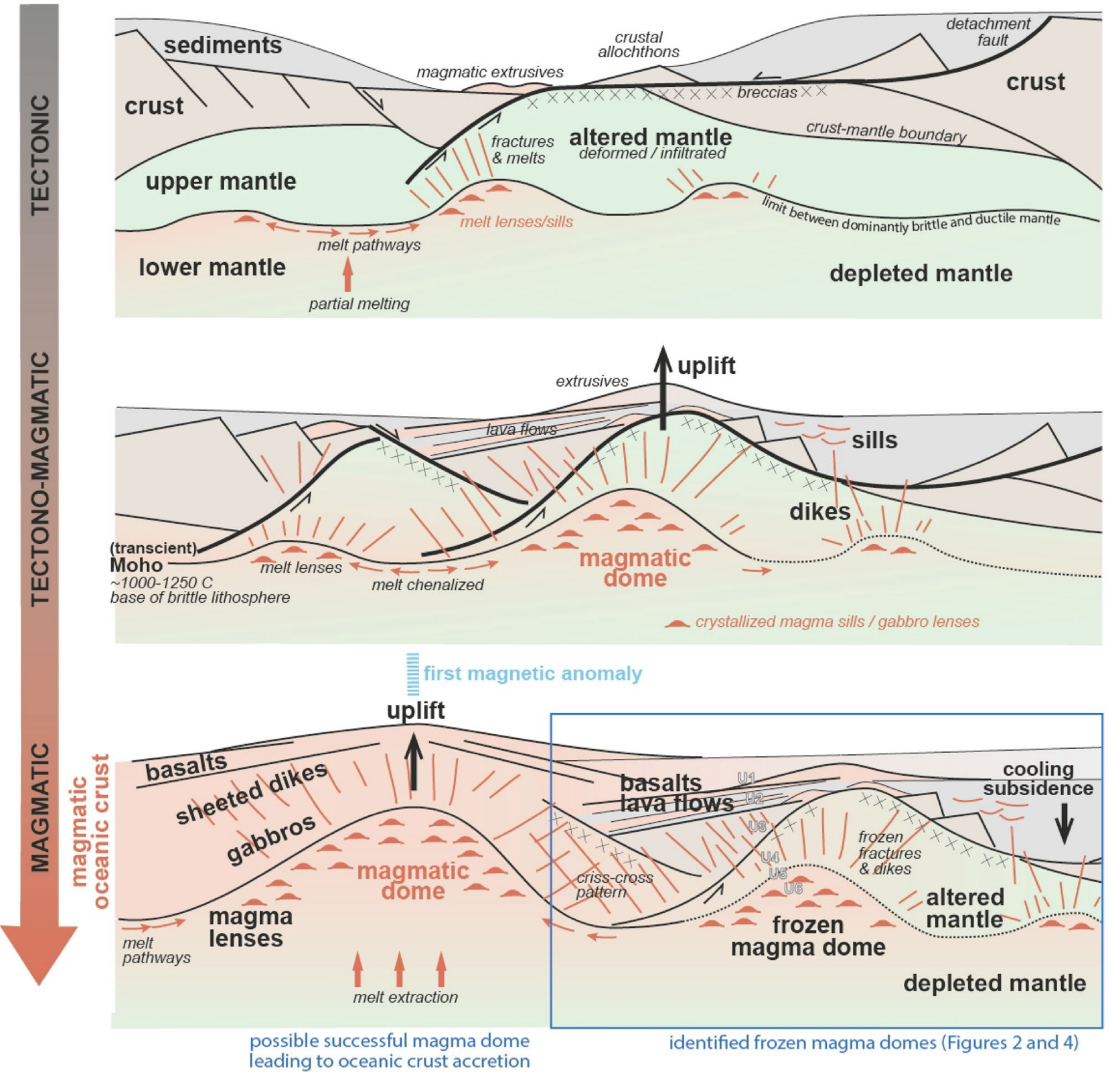
Schematic representation of the proposed evolution of the magmatic domes identified in the outer Møre and Vøring rifted margin. Please note that no attempt has been made to represent the water depth evolution of the area. See Discussion section for explanation.

Distal margin domains are often described as archiving the evolution from tectonic-dominated to magmatic-dominated extensional to accretional processes. Magma facilitates lithospheric thinning to occur at far lower stress levels than needed with faulting^57^^58^;. Thus, in theory, the genesis of magma in a rift system permits rifting at more modest stress levels, leading - gradually or abruptly - to a situation where and when the genesis of large-scale faults is not necessary anymore to accommodate extension. Within that framework, the magmatic domes identified here would tend to illustrate the evolution from the reign of the tectonic large-scale detachment faults in the rifted margin domain^59^ to the one of the magma dominance in the oceanic domain (Fig. 5) - the outer domain being the region where the transition occurs. The observations reported here show that this change was gradual in the case of the Mid-Norwegian rift system. Conversely, in other cases, the change can occur more rapidly, like in magma-rich environments.

Numerous interpretations and models have been proposed to characterize the magma-rich breakup (e.g^23^^60^^61^.,;;. Paton et al.^62^ examined the Argentine margin and found that the basement underlying the SDRs in the COTZ exhibits seismic characteristics similar to oceanic crust, with extrusive volcanics being significant. The authors proposed that this reflects fluctuating magma supply along an initiating spreading centre, similar to what is reported at active mid-ocean ridges^63^. Based on the same South Atlantic system, McDermott et al.^24^ pointed to the existence of actually 2 types of SDR, and proposed that the limit between the two would represent the transition from continental rifting (Type I SDR, planer, fault bounded) to the accretion of new magmatic crust (Type II SDR, convex-upward, subaerial or subsea). Another example of magma-rich environment can come from the South China Sea rift: this system shows important lateral structural and magmatic variations, including segments where the breakup has been magma-rich and the rift to drift change extremely abrupt, with intricate - and superposed - tectono-magmatic geometries^64^. Thus, in these two cases, the continent - ocean limit is interpreted as geographically well defined, although only (relatively) superficial geometries are reported.

In our Mid-Norwegian context, the question is to understand where a geographical limit should be interpreted to differentiate the continental vs. oceanic derived basement. If correctly interpreted, how/when and where the magmatic domes could evolve into a stable ocean spreading axis? Could these domes be the precursors of magma-chambers/crystal mush zones?

Spatially, on one profile, multiple domes can be observed (see Fig. 3a gridded surface), which suggests that several magmatic accumulations were initiated in the outer Norwegian margin. The observation is not dissimilar to some reports from oceanic ridge settings, like the Juan de Fuca Ridge that demonstrated the presence of multiple isolated magma bodies along a same ridge axis (e.g^49^^51^.,;. However, temporally, the evolution of these domes remains unconstrained. Each dome - if interpreted in terms of an accumulation of crystallized sills and/or gabbroic lenses - could represent one attempt at proto-ridge establishment, and the adjacent dome the next attempt, in a typical stuttering time evolution up to the genesis of more mature dome(s). An oceanward migration of the tectono-magmatic activity is often assumed in these distal rifted margin settings, where the deformation phases mostly migrate oceanward during the rift history. So, an interpretation assuming an oceanward migrating sequence of domes formation is highly tempting - as suggested for the conjugate North-East Greenland^65^. However, off-sequence attempts cannot be excluded. It has been demonstrated that some COTZs are characterized by tectonic activity that can regularly change dip direction, with exhumation detachment faults cutting through the previous geometries and redefining the upper plate vs. lower plate settings at the margin scale^66^^11^^67^;;. However, this observation is valid for tectonic geometries, what may not be the case for magmatic events.

Regarding the final establishment of the drift processes, it is assumed that the successful initiation of the oceanic spreading axis is determined by both local and regional constraints. Depending on some local parameters (e.g., rheology, spreading rate, magma supply, hydrothermalism, lithospheric stress), some magmatic domes may be more efficient than others in focusing and accumulating the magmatic melts. Then, the large-scale 3D geometrical configuration may lead to the lateral connection of some of the more mature domes. This would generate an axis of significant lithospheric weakening that can ultimately allow the successful start of the oceanic spreading ridge at the plate scale (Fig. 5).

These thoughts also relate to the definition of the ‘COB’ - Continent-Ocean Boundary. Like *breakup*, *COB* is a widely used term in the rift community. However, its definition is still ambiguous as its mapping is highly subjective and strongly dependent on the dataset^19^^9^;. For instance, the COB can be defined based on potential field grids - commonly magnetic anomalies. In that case, the definition depends on the interpreter’s judgement, on the quality of the dataset, and if breakup occurred when magnetic reversals were regular enough to permit a distinct record of the first accreted oceanic crust. The identification of the COB can alternatively be based on seismic data, defined by the first characteristic three-layers geometry attributed to normal Penrose-type oceanic crust. In this case, the identification very often suffers from the presence of salt or volcanics in the distal settings that can perturb the seismic signals. Additionally, this mapping strategy assumes the existence of an abrupt classical oceanic layered geometry, which may not necessarily be the case, depending on the magmatic supply and accretionary processes. Each method has advantages and disadvantages. The result, however, is that for a same margin, depending on the dataset and definition, interpreters can define different COBs, potentially up to dozens of km apart. Furthermore, the COB concept intrinsically assumes the existence of a sharp limit between the distal margin and the oceanic domain when it needs not to be the case. As discussed in this contribution, the transition from the margin to the ocean may display complex, interfingered and/or transitional geometries and lithologies - with no abrupt change. In these contexts, no strict line can be drawn between strict continental and oceanic rocks, as these can be many km apart. The definition of a boundary in these regions is probably unsuitable. Alternative mapping protocols should be investigated to capture the existence of accreted basement of different lithology than purely continental or oceanic.

It is noted that the observations listed here deserve extensive additional work, as it directly relates to the initiation and development of the ridge magmatic system. The water baselevel evolution is for instance a major open question that would need to be tackled, together with the SDRs subaerial emplacement and the related birth of the spreading system and its standard ~ 3500 m water depth. The lithospheric thermal state of the system is the other elephant in the room and need detailed investigations. If the magmatic domes reported here would be interpreted in terms of ridge axis ‘proto-magma chambers’/‘magma mush’, the thermal evolution of the related area would have been quite dramatic with periods of remarkable heating, with imprints that should still be observable. Future research works are presently designed to assess further these points.

## Conclusions

To conclude: the study reported here adds to our catalogue of observations related to the end of rifting - beginning of drifting. Dome-like geometries have been identified in the outer domain of the Mid Norwegian rifted margin and interpreted as probable crystallized magmatic bodies (gabbroic sills/lenses?) accumulated in the mantle. These domes are discussed as potential candidates for oceanic ridge stuttering initiation. An analogy with proto- magma chambers/magma mush is discussed. However, the thermal, spatial and temporal development of the magmatic domes remains unconstrained and need further investigation. In addition, to fully characterize the establishment of the ridge - and to properly constrain the relation between the domes reported here and the future ridge axis -, an extension of the dataset over the oceanic domain would be necessary.

### Methods

#### Dataset

The Geoex MCG Regional Deep Imaging 2019 (RDI19) dataset available for this study is a recent deep towed 16 s-twtt long offset seismic reflection regional dataset^68^. To image the deep basement rocks, such as the lower crust, Moho, and upper mantle, Geoex MCG focused on a long-offset acquisition setting with a large source of 6270 in^3^ and a 12 km long streamer with a continuous record of 16 s-twtt (seconds two-way travel time). The processing was designed to obtain the best imaging of the deep lithological units, including pre-stack noise and diffracted multiple attenuation, pre-migration noise attenuation, migration velocity model analysis, and pre-stack 2D Kirchoff time migration. With such acquisition and processing parameters, it is a unique dataset proposing an unprecedented imaging of the architecture of the Møre and Vøring segments of the Mid-Norwegian rifted margin. The full margin architecture is imaged for the first time including top basement, Moho and upper mantle, within all margin domains, from the most proximal regions in the East to the transition to the oceanic crust in the West (Fig. 1). Seismic interpretation has been performed under the Schlumberger Slb Petrel software.

## Limitations

Limitations regarding the work presented here are of two categories: data-related and interpreter-related.

Seismic interpretation is subjective, and multiple alternatives can be proposed. For a same dataset, opposing views can emerge, as for instance in the South Atlantic where the same COTZs crust can be interpreted as resulting from seafloor spreading by some authors (e.g^24^.,, and from the exhumation of middle-lower continental crust by others [e.g^69^., - what results in conflicting scenarios for the continental vs. oceanic domains evolution. Here, similarly, the proposed interpretation and discussion remains based on the sole understanding and background knowledge of the author. Recent interpretation experiments have highlighted the usefulness of compiling multiple interpretations to better evaluate the range of structural alternatives for a same dataset^16^. Such an exercise would certainly be interesting and useful to be run on the present region.

The data quality is another parameter of great importance. The GeoexMCG data used in this work is of highest quality, with the most recent acquisition and processing techniques. In the North Atlantic regions, the seismic reflection profiles are of good to excellent quality, imaging correctly the inboard geometries, but often struggle to image the most outboard features because of the presence of multiple shallow volcanics, lava flows, sills and intrusions that filter down the below seismic signals. The dataset used here is subject to the same drawbacks, however, its advantage is that the profiles are very long-offset and deep penetrating, what help illuminate the structures at depth below the outer high lava flows. Nevertheless, standard caution should always prevail when considering seismic features as some may be related to processing artefacts rather than real signal. For instance, the change in seismic character to the higher amplitude events reported here at depth is probably a robust observation. The systematic observation of the same geometries on all the profiles is also another strong point. On the other hand, the undulating geometry of the U5 interface can be questioned and may be associated with the stacking velocity model used at processing stage. This effect is known and taken into account in this study. It is recognized that although different velocity values will impact the geometry of the interface, potentially attenuating the domes vs. lows configuration, the marked undulating character will most probably remain and is thus considered as a valid geometry to be interpreted.

## Data Availability

The data that support the findings of this study are available from GeoexMCG but restrictions apply to the availability of these data, which were used under license for the current study, and so are not publicly available. Image data are, however, available from the author upon reasonable request and with permission of GeoexMCG.
